# *Arbuscular mycorrhiza* Symbiosis Induces a Major Transcriptional Reprogramming of the Potato *SWEET* Sugar Transporter Family

**DOI:** 10.3389/fpls.2016.00487

**Published:** 2016-04-14

**Authors:** Jasmin Manck-Götzenberger, Natalia Requena

**Affiliations:** Molecular Phytopathology, Botanical Institute, Karlsruhe Institute of TechnologyKarlsruhe, Germany

**Keywords:** SWEET transporters, potato, *Arbuscular mycorrhiza*, root, sugar transport, plants, symbiosis

## Abstract

Biotrophic microbes feeding on plants must obtain carbon from their hosts without killing the cells. The symbiotic *Arbuscular mycorrhizal* (AM) fungi colonizing plant roots do so by inducing major transcriptional changes in the host that ultimately also reprogram the whole carbon partitioning of the plant. AM fungi obtain carbohydrates from the root cortex apoplast, in particular from the periarbuscular space that surrounds arbuscules. However, the mechanisms by which cortical cells export sugars into the apoplast for fungal nutrition are unknown. Recently a novel type of sugar transporter, the SWEET, able to perform not only uptake but also efflux from cells was identified. Plant SWEETs have been shown to be involved in the feeding of pathogenic microbes and are, therefore, good candidates to play a similar role in symbiotic associations. Here we have carried out the first phylogenetic and expression analyses of the potato SWEET family and investigated its role during mycorrhiza symbiosis. The potato genome contains 35 SWEETs that cluster into the same four clades defined in *Arabidopsis*. Colonization of potato roots by the AM fungus *Rhizophagus irregularis* imposes major transcriptional rewiring of the SWEET family involving, only in roots, changes in 22 of the 35 members. None of the SWEETs showed mycorrhiza-exclusive induction and most of the 12 induced genes belong to the putative hexose transporters of clade I and II, while only two are putative sucrose transporters from clade III. In contrast, most of the repressed transcripts (10) corresponded to clade III SWEETs. Promoter-reporter assays for three of the induced genes, each from one cluster, showed re-localization of expression to arbuscule-containing cells, supporting a role for SWEETs in the supply of sugars at biotrophic interfaces. The complex transcriptional regulation of SWEETs in roots in response to AM fungal colonization supports a model in which symplastic sucrose in cortical cells could be cleaved in the cytoplasm by sucrose synthases or cytoplasmic invertases and effluxed as glucose, but also directly exported as sucrose and then converted into glucose and fructose by cell wall-bound invertases. Precise biochemical, physiological and molecular analyses are now required to profile the role of each potato SWEET in the arbuscular mycorrhizal symbiosis.

## Introduction

Plants are the major factories of reduced carbon on the earth and therefore most of the other living organisms are absolutely dependent on them. This statement is particularly true for obligate biotrophic microorganisms that feed on living plants and are, thus, committed to modify the plant cell program toward the release of sugars into the extracellular space. It is not long ago that the molecular mechanisms of how plants release sugars to the extracellular space were unknown, and thus, the identification of SWEET transporters as key players in this process represented a landmark (Chen et al., 2010).

SWEET transporters are integral membrane proteins with seven transmembrane domains that cannot only catalyze the efflux of carbohydrates but also their uptake (Chen et al., 2010; Chen, 2014). SWEETs were discovered in a screening using FRET glucose or sucrose sensors to identify pH independent sugar transporters in *Arabidopsis* (Chen et al., 2010, 2012; Lin et al., 2014). They are though very conserved throughout the plant kingdom, and Angiosperms contain a large number of *SWEET* genes, in average 20 according to Eom et al. (2015), but up to 52 in soybean (Patil et al., 2015). Phylogenetic analyses in several plants have shown that SWEETs can be grouped in 4 clades that were first defined in *Arabidopsis* (Chen et al., 2010; Chong et al., 2014; Wei et al., 2014; Feng et al., 2015; Patil et al., 2015). Assignment to one clade seems to correlate with the substrate transported rather than with the physiological function exerted by the SWEETs (Eom et al., 2015). Thus, from the functional analyses mainly carried out in *A. thaliana* and rice, it appears that SWEETs in clades I and II preferentially transport hexoses, while the characterized SWEETs from clade III are sucrose transporters (Chen et al., 2010, 2012; Lin et al., 2014). Clade IV contains less SWEET genes than the other clades and they have been shown to be vacuolar transporters controlling the flux of fructose across the tonoplast (Chen et al., 2010; Chardon et al., 2013; Guo et al., 2014).

SWEETs serve many different functions in plants, including the export from the phloem parenchyma cells in source tissues previous to phloem loading by SUT/SUC importers, pollen nutrition by the tapetum, embryo development or nectar secretion (Ge et al., 2000; Chen et al., 2010, 2012, 2015b; Lin et al., 2014). Therefore, it is not surprising that if the efflux of sugars from cells requires the participation of SWEET transporters, microbes feeding on living plant cells manipulate their expression to increase sugar release. And indeed, several rice SWEET promoters have been shown to be the target of several TAL (transcriptional activator-like) effectors from the pathogenic bacteria *Xanthomonas oryzae* pv. *oryzae* (Chu et al., 2006; Yang et al., 2006; Antony et al., 2010; Chen et al., 2010; Streubel et al., 2013). The responsible TAL effectors delivered into the plant cytoplasm by means of the type III secretion system reach the nucleus to induce the expression of specific SWEETs, guaranteeing sucrose delivery into the apoplast of colonized cells. Although, so far no other examples of the mechanisms of ectopic SWEET induction by microbes have been elucidated, it is known that many microorganisms including symbiotic bacteria, fungi and oomycetes induce SWEET expression during plant colonization (Gamas et al., 1996; Yu et al., 2010; Chong et al., 2014; Chen et al., 2015a). Thus, it is tempting to speculate that convergent evolution might have made the promoter of SWEETs a very attractive target for biotrophic organisms. This is supported by the findings of Streubel et al. (2013) who proved that the promoter of the rice OsSWEET14 contains binding sites for at least four different TAL effectors. But also by the results of Cohn et al. (2014) showing that not only in rice, but also in the dicot cassava, SWEET promoters are the target of TAL effectors from *X. axonopodis* pv. *manihotis*.

The symbiotic AM fungi are well known for their beneficial effects on plants (Smith and Smith, 2011). These soil fungi from the phylum Glomeromycota colonize the cortex of plant roots and establish a mycelial network in the soil that allows plants to access scarce mineral nutrients such as phosphorous. In turn, mycorrhizal plants provide AM fungi with carbon, as they are obligate biotrophs and can only complete their life cycle in symbiosis (Smith and Smith, 2012). Soil phosphate is transported along the mycelial network into the inner cortex of the root, where it is delivered at specialized fungal structures called arbuscules that also serve for the sugar uptake from the plant. Arbuscules are tree-like structures formed by profuse dichotomous hyphal branching in the lumen of cortical cells surrounded by a plant derived plasma membrane called the periarbuscular membrane (PAM). The PAM is very special as it has a different protein composition from the rest of the plasma membrane, hosting the transporters that will be key in the nutrient exchange with the fungal partner (Pumplin and Harrison, 2009; Zhang et al., 2015). Plants forming AM symbiosis have a dramatically different physiology, as they are not only better provided with mineral nutrients but also have an increased sink strength toward the root that imposes a carbon partitioning reallocation (Wright et al., 1998; Graham, 2000; Boldt et al., 2011). The form in which carbon is provided from the host plant to the AM fungus is still under debate, although most of the experimental evidence suggests that glucose is the main form taken up by the fungus (Shachar-Hill et al., 1995; Solaiman and Saito, 1997; Pfeffer et al., 1999). However, the finding that AM fungi lack the enzyme for *de novo* fatty acid biosynthesis, the type I FAS, (Tisserant et al., 2013; Salvioli et al., 2016; Tang et al., 2016) led to the hypothesis that, in addition to sugars, plants might be providing lipids as a source of carbon to their symbionts (Wewer et al., 2014). Currently, however, there is no experimental evidence on the mechanisms of how fatty acids could be released into the periarbuscular space or taken up into the fungus. In contrast, molecular support for sugar import into the fungus from the apoplastic space was obtained with the identification of the high affinity monosaccharide transporter MST2 from the AM fungus *Rhizophagus irregularis*, only expressed in symbiosis at arbuscule containing cells and intercellular hyphae (Helber et al., 2011). MST2 can transport several monosaccharides, including some pentoses, but it has the greatest affinity for glucose. Its inactivation by HIGS (host induced gene silencing) severely impaired arbuscule formation suggesting that this transporter is critical for the symbiosis (Helber et al., 2011).

The key questions are, however, how does glucose reach the apoplastic space in colonized cells? And, how does the plant regulate the release of sugars into the apoplast? Enzymatic and promoter-reporter assays have clearly shown that mycorrhizal roots have a significant increase in cell wall-bound (CW) invertase activity (Wright et al., 1998; Schaarschmidt et al., 2006). Furthermore, CW invertase activity is located in the apoplast surrounding arbuscule-containing cells and intercellular hyphae, suggesting that apoplastic sucrose is cleaved prior uptake by the fungus (Schaarschmidt et al., 2006). Sucrose is the main form in which sugars are transported toward sink tissues including the root. However, after leaving the vascular tissue at the root, sucrose has to go through the endodermis to reach the cortical cells. Because sucrose is then expected to move symplastically to overcome the casparian strip (Kaiser et al., 2014), the involvement of SWEET exporters in cortical cells containing arbuscules is anticipated. In order to test this hypothesis, we decided to characterize the SWEET family of transporters in the mycorrhizal plant *Solanum tuberosum* (hereafter potato).

Here we show that potato contains a large SWEET family with 35 members, and that mycorrhiza colonization imposes a major transcriptional regulation of SWEETs in roots. The promoter activity of three up-regulated SWEETs during mycorrhiza colonization was analyzed using the GUS reporter gene and showed highest induction in arbuscule-containing cells. Altogether, our results point toward an important role for SWEET transporters during the mycorrhiza symbiosis.

## Materials and methods

### Plant material and growth conditions

*Solanum tuberosum* cv. Desiree was propagated as cuttings axenically in plastic containers with Murashige and Skoog medium with vitamins and 25 g/l sucrose (Murashige and Skoog, 1962) solidified with 1 g/l Phytagel (P8169, Sigma-Aldrich, Germany; http://www.sigmaaldrich.com/germany.html) at 21°C and 16/8 h day/night rhythm.

For the tissue analysis, 2 week old cuttings were transferred to 9 cm/500 ml pots with a sand:gravel (1:4) mixture. The plants were fertilized once a week with 50 ml half strength Long Ashton nutrient solution with high phosphate content (665 μM; Hewitt, 1966). After 3 weeks at 23°C and 16/8 h day/night rhythm, roots, stems and leaves were harvested separately for RNA extraction. Five plants from independent pots were pooled to form one biological replicate, three biological replicates were used. For the harvest of tubers, plants were grown until tuberisation.

For mycorrhiza colonization, potato cuttings were transferred to pots as described above and adapted for 1 week to those conditions (23°C). After that, they were inoculated by mixing the substrate with 2 months old *Daucus carota* hairy root cultures grown monoxenically in association with *Rhizophagus irregularis* DAOM 197,198 (Schenck and Smith, 1982; Krüger et al., 2012) on M-medium with sucrose at 27°C in darkness (Bécard and Fortin, 1988). One Petri dish of carrot roots was used to inoculate a 500 ml pot. Plants were fertilized with half strength Long Ashton nutrient solution with 5 μM phosphate. Non-mycorrhizal controls were treated the same. Roots were harvested 4, 6, and 8 wpi (weeks post inoculation) for RNA extraction. Three plants from independent pots were pooled to form one biological replicate. Three biological replicates per treatment were used.

*Medicago truncatula* Jemalong A17 was transformed as described using *Agrobacterium rhizogenes* ARquaI (Kuhn et al., 2010). Five weeks after transformation, wild type roots were cut and the plants were propagated one additional week on medium supplied with 400 mg/l Augmentin (AmoxiClav, Hikma Farmaceutical, Portugal; http://www.hikma.com/). After that, the composite plants were transferred to 50 ml growth tubes (Stuewe & Sons, Inc., USA; https://www.stuewe.com/) with the same substrate described above which was directly inoculated with mycorrhizal carrot roots (1/4 Petri dish per 50 ml tube). They were fertilized with 5 ml half strength Long Ashton nutrient solution (low phosphate, 20 μM) once a week and harvested at 5 wpi.

### Identification of *SWEET* genes, phylogeny, and synteny analysis

*SWEET* genes were identified via BLASTP search in the National Center for Biotechnology Information (NCBI; http://www.ncbi.nlm.nih.gov/) and the Solanaceae Genomics Network database (SGN; Bombarely et al., 2011; http://solgenomics.net/). The 35 SWEET genes identified were named *S. tuberosum SWEET1* to *SWEET*17. Numbers were given according to their closest homolog of *Arabidopsis thaliana* (Chen et al., 2010). For numbers that were present more than once small letters from a to g were assigned according to their positions in the genome (Supplementary Table 1).

Phylogenetic analyses were conducted using Clustal Omega (Sievers et al., 2011, https://www.ebi.ac.uk/Tools/msa/clustalo/) for protein sequence alignment and Mega 6 (Tamura et al., 2013, http://www.megasoftware.net/) for the phylogenetic tree construction. The Neighbor-joining method with the p-distance substitution model was applied, performing Bootstrapping with 1000 replicates and missing data was deleted pairwise. The clades were classified according to Chen et al. (2010). The accession numbers of the sequences used can be found in Supplementary Table 1 and were obtained from NCBI, the *Arabidopsis* Information Resource (TAIR; Lamesch et al., 2012; https://www.*Arabidopsis*.org) or from the SGN. For synteny analysis, the genome positions of the potato *SWEETs* (Supplementary Table 1) and the positions of the corresponding tomato homologs were used (Feng et al., 2015).

### Topology prediction and manual annotation

The transmembrane helices of all SWEETs were predicted by TMHMM Server v.2.0 (http://www.cbs.dtu.dk/services/TMHMM/). For those not showing the typical seven transmembrane domain structure, a manual annotation was carried out using BLASTX in NCBI as well as by using cDNA analyses. The number of transmembrane helices of the final predicted proteins can be found in Supplementary Table 1. All the potato sequences used in this paper were sent to NCBI and are available with GenBank accession numbers KU686963 to KU686997 (Supplementary Table 1).

### Expression data and quantitative real-time expression analyses

Expression data of RNA-seq experiments from the Potato Genome Sequencing Consortium was shown as fragments per kilobase of transcript per million mapped reads (FPKM). Values were obtained from the Spud DB Solanaceae Genomics resource (Hirsch et al., 2014; http://solanaceae.plantbiology.msu.edu/) for those SWEETs having a potato genome annotation. Nine of the 35 potato *SWEET* genes are currently not annotated in the potato genome and therefore, their expression was analyzed by RT-PCR with 40 cycles (see primer list Supplementary Table 2). Total RNA was extracted using the innuPREP RNA Kit (Analytik Jena AG, http://www.analytik-jena.de/), quantified by the NanoDrop ND-100 spectrophotometer (http://www.nanodrop.com/). cDNA was synthesized as described elsewhere (Kuhn et al., 2010) with the reverse transcriptase SuperScript II (Invitrogen, USA; https://www.lifetechnologies.com/de/de/home.html).

Real time expression analyses were carried out using an iCycler MyIQ (Bio-Rad, USA; http://www.bio-rad.com/) and MESA Green 231qPCR Master Mix Plus (Eurogentec, Germany; http://www.eurogentec.com/eu-home.html) with three technical replicates per reaction and three independent biological replicates. Expression of the translation elongation factor 1-alpha (*Stef1*; AJ536671) was used for normalization. *StPT4* (AY793559), *StInvCD141* (Z22645), *RiTEF* (DQ282611), and *RiMST2* (HM143864) served as indicators of symbiosis status. Primers used can be found in Supplementary Table 2.

### Promoter analysis

Promoter elements were searched in the 2 kb region upstream of the ATG for each SWEET gene from potato and from tomato (see Supplementary Table 3 for sequence details). For *StSWEET7d, StSWEET12c, StSWEET12e, SlSWEET3*, and *SlSWEET12a*, only smaller fragments could be obtained from the databases (1491, 1207, 1900, 1235, and 863 bp, respectively).

For promoter-reporter assays, 2 kb fragments of the *SWEET2c, SWEET7a*, and *SWEET12a* promoters were cloned into the Gateway binary vector pPGFPGUS-RR, containing the reporter genes GFP (green fluorescent protein) and GUS (β-Glucuronidase) as well as a red root cassette (RR, constitutively expressed DsRed) for root transformation control (Kuhn et al., 2010). *A. rhizogenes*-mediated transformation of *M. truncatula* and mycorrhizal inoculation with *R. irregularis* was carried out as described above. GUS staining was performed as described elsewhere (Kuhn et al., 2010) with the following modifications: the staining was fixed with 50% EtOH for 2 h. After that, the roots were cleared in 10% KOH for 30–45 min at 95°C and fungal cell walls were stained with WGA-FITC (wheat germ agglutinine-fluorescein isothiocyanate, Sigma Aldrich) according to Rech et al. (2013). For each construct, at least 10 independently transformed roots were analyzed.

### Microscopy and image processing

Roots expressing GUS were microscoped using a Leica TCS SP5 (DM5000) equipped with the digital color camera DFC295. Objectives used were HC PL FLUOTAR 10.0 × 0.03 DRY, HCX PL APO CS 20.0 × 0.70 DRY UV, and HCX APO lambda blue 63.0 × 1.20 WATER UV CORR (Leica, Wetzlar, Germany; http://www.leica-microsystems.com/de/) at 21°C. Differential interference contrast (DIC) pictures were obtained using appropriate DIC prisms for the three objectives and FITC fluorescence was recorded using filter cube L5. Images were collected using LASAF v2.6 and ImageJ 1.48p (http://fiji.sc/Fiji).

### Statistical analyses

Expression analysis data were statistically tested for differences between non-mycorrhizal and mycorrhizal roots (three biological replicates, respectively) using the Student's *t*-Test with a two-tailed distribution and homoscedasticity. Differences were considered as significant with a value of *p* < 0.05 (marked with ^*^) and *p* < 0.01 (marked with ^**^).

## Results and discussion

### Identification and phylogenetic analysis of the SWEET family in potato

**Figure 1:**
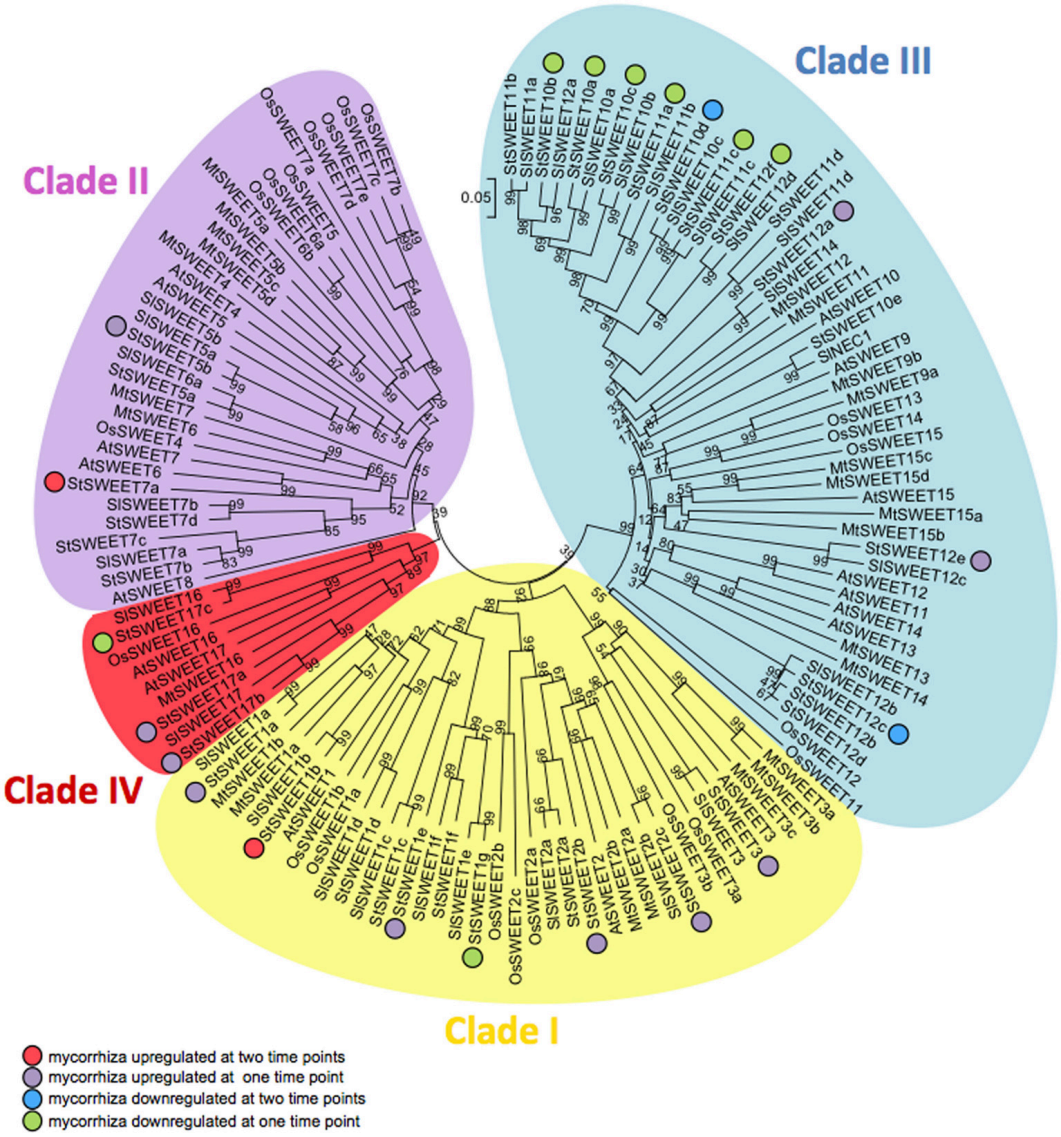
**Phylogenetic tree of potato, tomato, rice, ***M. truncatula***, and ***A. thaliana*** SWEETs**. The unrooted tree was generated based on an amino acid alignment using Clustal Omega. The phylogenetic tree was constructed using the neighbor-joining method and the p-distance model using MEGA 6. Scale bar represents evolutionary distance in number of amino acid differences per site. Bootstrapping was performed with 1000 replicates and values are displayed on branches in %. Colored circles represent regulation by mycorrhization in the same color code as in **Figure 5**. Gene names and accession numbers can be found in Supplementary Table 1.

In order to get insights into the mechanisms of sugar homeostasis in roots during mycorrhiza formation and in particular into the role of SWEETs, we first carried out *in silico* sequence analyses in the genome of potato (*S. tuberosum* group Phureja). Following the comprehensive phylogenetic analysis that was carried out in the model plant *Arabidopsis thaliana* (Chen et al., 2010), we identified 35 gene sequences coding for putative SWEETs using BLASTP searches in NCBI as well as in the SGN (Sol Genomics Network) databases (Figure 1). This number is much higher than the average number of SWEETs for angiosperms (*ca*. 20) (Eom et al., 2015) perhaps reflecting the genome triplications and gene tandem duplications observed in the *Solanum* lineage (Xu et al, 2011; Sato et al., 2012). We named the potato SWEETs following the *A. thaliana* nomenclature (Chen et al., 2010). Protein alignment of the 35 potato SWEETs with 29 sequences from tomato, 17 from *A. thaliana*, and 21 from rice allowed the construction of a phylogenetic tree. All 35 potato SWEETs cluster into the four clades defined by Chen et al. (2010). Most of the SWEETs from potato belong to clade III (15), followed by SWEETs from clades I and II (11 and 6, respectively). In addition, potato has also three SWEETs in the clade IV, whose members have been shown to transport fructose across the tonoplast (Chen et al., 2010; Chardon et al., 2013; Guo et al., 2014).

Interestingly, in tomato only 29 SWEET transporters have been identified (Feng et al., 2015). This reduction is homogenously distributed among the clades. The overall identity of all potato SWEETs is *ca*. 41%, while the overall identity of all SWEETs depicted in the phylogenetic tree of Figure 1 is 39%, indicating a high degree of conservation among SWEET proteins. Pairwise comparison of potato and tomato SWEETs revealed that some members are up to 97% identical, being the overall identity between tomato and potato of ca. 42%, mirroring the overall similarity between the two genomes (Sato et al., 2012; Supplementary Table 4).

**Figure 2:**
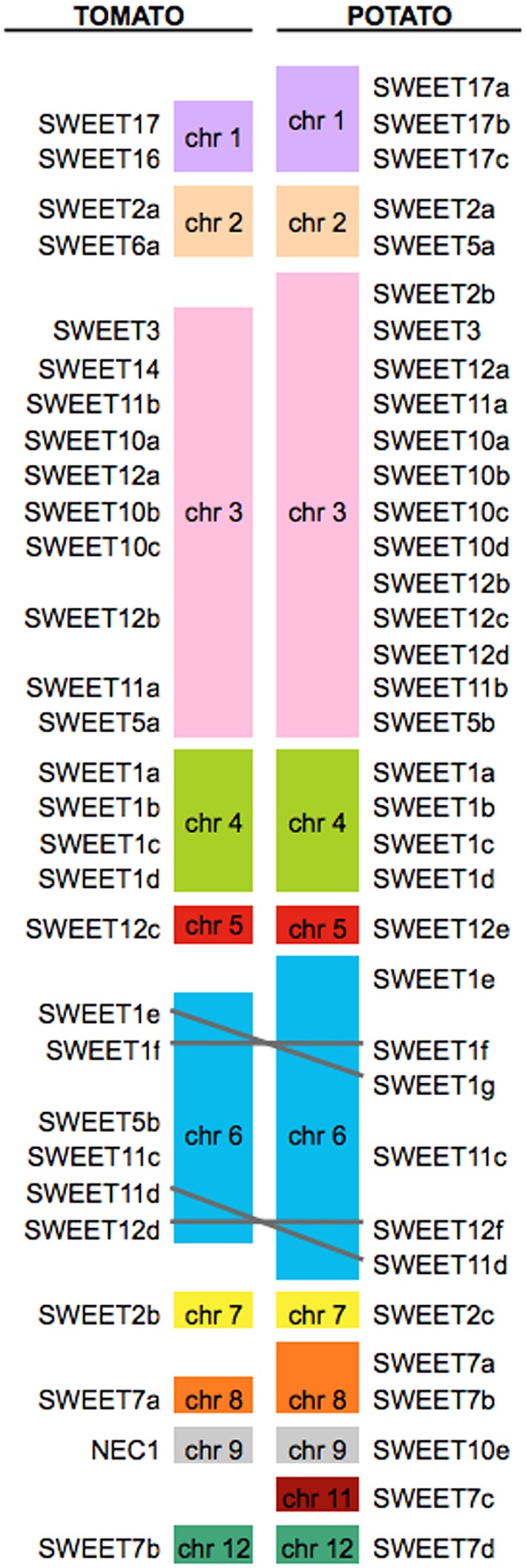
**Synteny analyses of ***SWEET*** genes in potato and tomato**. *SWEET* genes are present in almost all chromosomes with the exception of chromosome 10 in potato and 10 and 11 in tomato. The distribution of the genes in both genomes is almost identical, with only two inversions in chromosome 6, mirroring the general high degree of synteny between both genomes.

The organization of genes encoding SWEETs in the potato genome showed that they are widely distributed among 11 from the 12 chromosomes (Figure 2). Chromosome 3 encodes the highest number of SWEETs (13 members), while chromosome 10 contains no *SWEET* genes. The expansion of SWEETs in chromosome 3 seems to be Solanaceae specific as they are absent in *Arabidopsis*, rice and *Medicago*. In support of a specific function in Solanaceae, expression data in tomato have shown a common regulation for six of those genes (Feng et al., 2015). One partial *SWEET* gene (XM_006368013) was not ascribed to any chromosome, however it is likely located on chromosome 5 because manual annotation and expression analyses showed that together with the partial *SWEET* gene XM_006359017 located on chromosome 5, both comprise one single gene (see topology predictions below). Our synteny analysis shows that all potato *SWEET* genes having an ortholog in tomato are located in the same chromosome in both species (Figure 2), consistent with the global synteny observed between the two genomes (Sato et al., 2012). Seven potato *SWEET* genes do not have orthologs in tomato, while only one tomato gene misses an ortholog in potato. The synteny analysis showed that most potato *SWEET* genes are located in the same arrangement as in the tomato chromosomes, with only two inversions observed in chromosome 6 (Figure 2). All *SWEET* genes encoding transporters from clade IV are located in chromosome 1 in tandem, whereas members of the other clades are not specifically associated to any particular chromosome.

The discovery of SWEETs as sugar transporters was seminal because thus far only proteins with 12 transmembrane domains (TMs) or 14 TMs had been shown to be able to transport monosaccharides and disaccharides across membranes (Chen et al., 2010). In contrast, plant SWEETs were shown to contain only seven TMs and to form a structure with two tandem triple helix bundles (TM1-3) and (TM5-7) separated by a linker region including a less conserved TM (TM4). This structure allows the formation of two pseudosymmetrical halves that permits sugar passage through the membrane (Tao et al., 2015). Interestingly, bacterial SemiSWEETs contain only three TMs and are thought to achieve sugar transport by oligomerizing with other SemiSWEETs and are, therefore, considered ancestor proteins of eukaryotic SWEETs (Xuan et al., 2013; Wang et al., 2014; Xu et al., 2014; Tao et al., 2015). Surprisingly topology predictions for the potato SWEETs using TMHMM revealed that although most of them belong to the seven TM groups, several exceptions existed and 12 potato SWEETs did not conform to this pattern according to the annotations from NCBI and SGN (Supplementary Figure 1). However, a closer look to their sequences and expression analyses (data not shown) revealed that in most cases those genes and their proteins were wrongly annotated. Only one protein seems not to conform to the seven TMs pattern (SWEET7c), that only contains the first four TM domains. However *SWEET7c* seems to be a pseudogene because downstream of the domain coding for the fourth TMs another gene is located coding for an F-box protein (XM_006356285.2, F-box protein At2g32560-like). A putative correct annotation for all potato *SWEET* genes has been submitted to the NCBI database (Accessions KU686963 to KU686997).

### Tissue expression analyses of potato SWEETs

**Figure 3:**
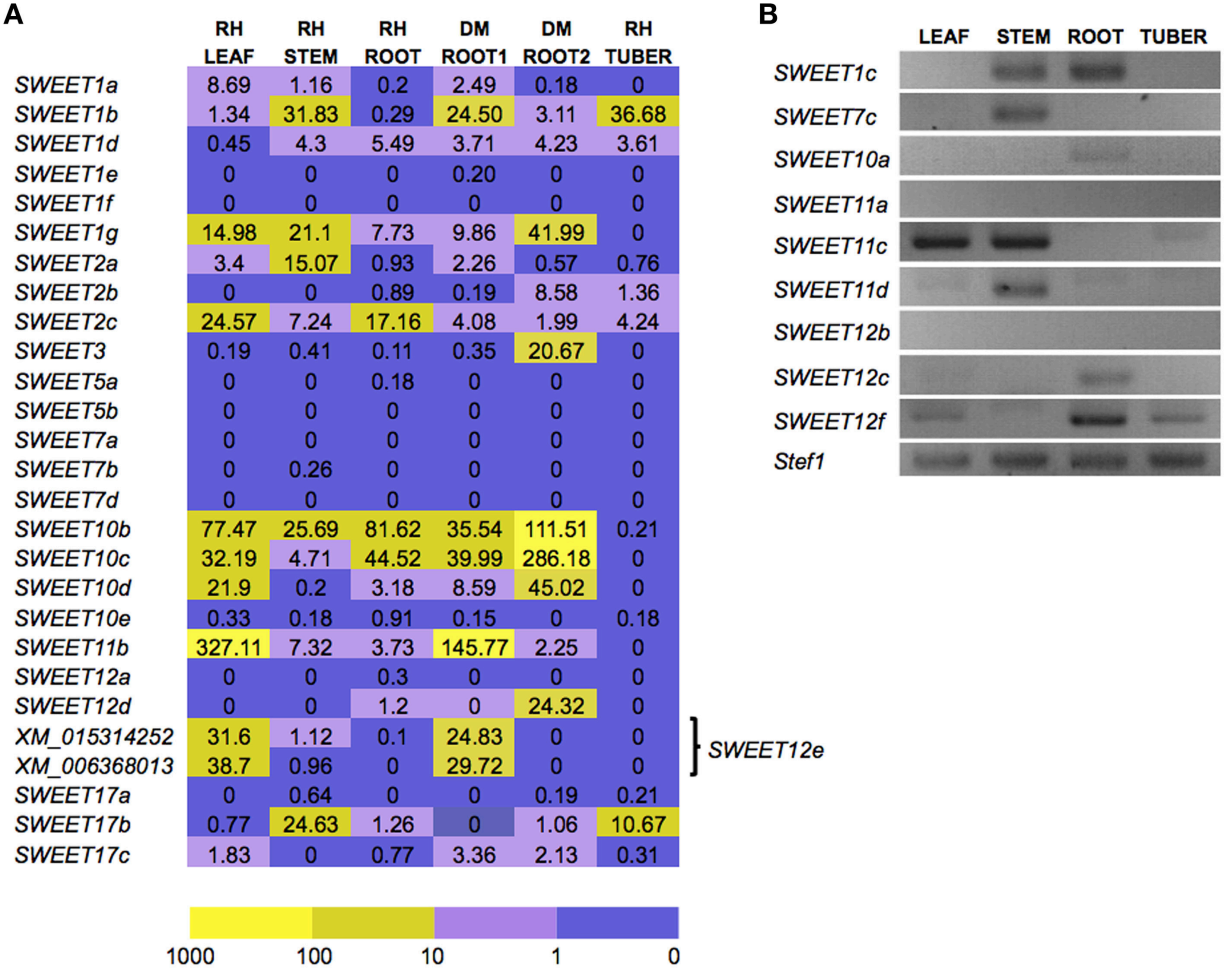
**Expression analyses of potato ***SWEET*** genes. (A)** *In silico* expression analysis of 26 of the 35 potato *SWEET* genes in several tissues, including flower, leaf, stem, root, stolon, and tuber from either RH89-039-16 (RH) or DM3-1 (DM), according to the RNA-seq data from the Spud DB expressed as FPKM values (fragments per kilobase of transcript per million mapped reads). Two independent root expression data sets are available for DM root (DM ROOT1, DM ROOT2). Since *SWEET12e* was not correctly annotated prior to this work, two transcripts can be found corresponding to this gene. The color code indicates level of expression in logarithmic scale from dark purple to bright yellow. **(B)** RT-PCR expression analyses in leaf, stem, root, and tuber for the nine potato *SWEET* genes not annotated in the SGN database. The house keeping gene elongation factor 1α (*Stef1*) was used as control. Forty cycles were used for the PCR.

We next analyzed *in silico* the transcriptional profile of potato *SWEET* genes using the Spud DB, comparing several tissues (Xu et al, 2011; Hirsch et al., 2014), mainly focusing on the expression profiles in roots. Nine from the 35 *SWEET* genes are not present in the SGN database and are therefore missing from the Spud DB. We analyzed those using RT-PCR in leaf, stem, root and tuber tissues from the potato cultivar Desiree. Results from the RNA-seq data in Spud DB showed that some of the *SWEET* genes were almost not detectable in most of the tissues analyzed such as *SWEET1f*, a few others were only expressed in one or two tissues. Thus, *SWEET7a* and *7d* are almost exclusively expressed in fruit, *SWEET1e and 5b* mainly in flower, and *SWEET12a* and *5a* in fruit and flower (Figure 3A, Supplementary Table 5). The only *SWEET* gene expressed almost exclusively in root is, according to Spud DB, *SWEET12d*. However, FPKM values are in general relatively low, and thus care must be taken when interpreting their meaning. From the 8 *SWEET* genes analyzed by RT-PCR, only *SWEET10a* and *12c* appeared as root specific, whereas *SWEET7c* and *11d* showed stem-specific expression (Figure 3B). However, we did not analyze tissues such as flower or stolons and therefore, more in depth expression analysis should be required. In general, the highest expression levels were found in leaf tissue, while mature tuber showed very low levels of expression for all *SWEET* genes, with the exception of *SWEET1b*. The two most expressed genes are *SWEET11b* in leaf and *SWEET1b* in petals, followed by *SWEET10b* in one of the root samples. These results show that although some SWEETs have been shown to have a specific function in some tissues, the high level of conservation in sequence and in expression anticipates a large degree of functional redundancy in potato.

### Transcriptional regulation of potato *SWEET* genes during mycorrhiza symbiosis

**Figure 4:**
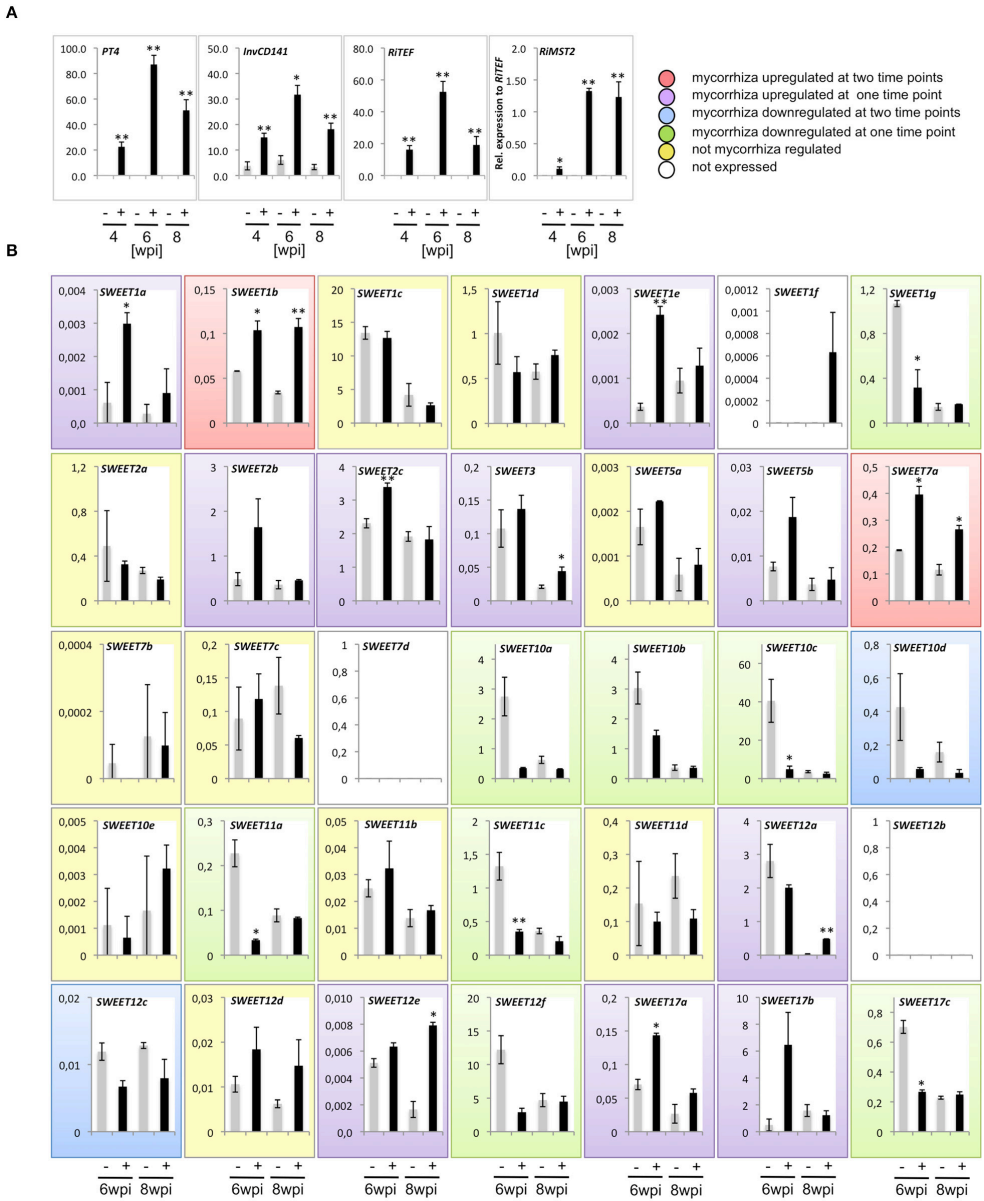
**Expression analysis of all potato ***SWEETs*** in roots in response to colonization by the ***Arbuscular mycorrhizal*** fungus ***R. irregularis***. (A)** The expression of the phosphate transporter *PT4* and the cell wall-bound invertase *InvCD141* from potato, as well as the fungal translation elongation factor 1a *RiTEF* and the monosaccharide transporter gene *RiMST2* were analyzed at 4, 6, and 8 weeks post inoculation (wpi). **(B)** Expression of potato *SWEETs* was measured at 6 and 8wpi. Expression is shown as relative expression to potato elongation factor 1α (*Stef1*) or to *RiTEF* for *RiMST2*. Error bars represent standard error of the mean. Non colonized samples are indicated by minus (−) and colonized samples by plus (+), per treatment the average expression of three biological replicates is shown. Student's *t*-test was used to calculate significance of mycorrhized compared to non mycorrhized samples in the same time point (^**^p < 0.01, ^*^p < 0.05).

To investigate the possibility that SWEETs are involved in sugar downloading processes during AM symbiosis, transcriptional changes in the expression of all potato SWEETs were analyzed in colonized roots. Potato plants were inoculated with the fungus *R. irregularis* and harvested at three time-points (4, 6, and 8 weeks post inoculation, wpi). To assess the development of the symbiosis, we first measured the expression of *StPT4* (an arbuscule-specific plant phosphate transporter) and of *RiTEF* (*R. irregularis* Translation elongation factor 1a). The level of *RiTEF* indicates the level of fungal colonization within the roots, while the plant phosphate transporter *StPT4* is only located in functional arbuscular branches and therefore it is a good indicator of active symbiosis (Harrison et al., 2002). Furthermore, we also analyzed the expression of the *InvCD141* gene, encoding a CW invertase whose ortholog in tomato is induced during AM symbiosis around arbuscules and intercellular hyphae (Schaarschmidt et al., 2006). In addition, we analyzed the expression of the fungal monosaccharide transporter *MST2* from *R. irregularis* (*RiMST2*) induced during symbiosis (Helber et al., 2011). While *StPT4, RiTEF*, and *RiMST2* were almost exclusively expressed in mycorrhizal samples as expected, the CW invertase showed also expression in non-colonized roots, although fungal colonization significantly raised its expression levels (Figure 4A). This result suggests that root colonization by *R. irregularis* caused an increase in apoplastic sucrose, contributing to increase the sink strength of mycorrhizal roots. The expression of all genes was highest at 6 wpi indicating that this was likely the time-point at which the symbionts were more active at nutrient exchange (Figure 4A).

We then analyzed the expression of all *SWEET* genes at 6 and 8 wpi. No transcript, or only barely measurable expression, was observed for three of the genes analyzed (*SWEETs 1f, 7d*, and *12b*), in general in accordance with the data from Spud DB (Figure 4B, Supplementary Table 5). In contrast, we could measure expression for 4 *SWEET* genes that had not been previously detected or only at very low levels in the Spud DB. Thus, *SWEET1e, 5b*, and *17a* showed expression, albeit low, in control roots and were induced in response to fungal colonization, mainly at 6 wpi, coinciding with the highest symbiotic activity (Figures 4A,B). Similarly, *SWEET7a* was also expressed in control roots, and induced even further by *R. irregularis* at both time-points (Figure 4B).

Remarkably, from all 35 potato *SWEET* genes, none of them showed a mycorrhiza-restricted induction, although two of them were clearly induced during symbiosis at 6 and 8 wpi (*SWEETs 1b* and *7a*), and 10 were induced at least in one time point (*SWEETs 1a, 1e, 2b, 2c, 3, 5b, 12a, 12e, 17a*, and *17b*). Interestingly analysis of expression data in the MtGEA (http://mtgea.noble.org/v3/) showed that several *SWEETs* were also induced during mycorrhiza symbiosis in *Medicago truncatula*. Thus, *MtSWEET1b, MtSWEET6*, and *MtSWEET9b* showed highest expression in arbuscule-containing cells, coincident with their putative potato relatives (*StSWEET1a, 1b, 7a*, and *12a*). In rice, analyses of expression in mycorrhizal roots only showed a clear induction for *OsSWEET3b* (Fiorilli et al., 2015), that also matches with the mycorrhizal induction of *StSWEET3*. These results suggest that several SWEETs might have been recruited for mycorrhiza symbiosis early in plant evolution.

*Arbuscular mycorrhizal* colonization also led to downregulation of some of the transporters. Thus, the expression of two of them (*SWEETs 10d* and *12c*) was repressed at both time-points, while 7 of them (*SWEETs 1g, 10a, 10b, 10c, 11a, 12f*, and *17c*) showed repression at 6 wpi (Figure 4B). Data from the MtGEA only showed a clear downregulation in arbuscule-containing cells for MtSWEET16 that cluster within the clade IV of tonoplast transporters. In contrast, RNA-seq data from rice only showed downregulation of *SWEET16* when comparing large lateral roots, which become colonized by mycorrhiza, vs. fine lateral roots that are not (Fiorilli et al., 2015).

Remarkably, 7 of the 10 repressed potato SWEETs belong to what appears to be a Solanum-specific subclade within clade III. Interestingly, the orthologs of five from them in tomato were shown to be repressed in roots in response to different stresses including sugars, temperature and salt, while at the same time induced by the same stresses in leaves (Feng et al., 2015). These results suggest that such group of genes plays a role in the carbon allocation between roots and shoots in tomato in response to external stimuli. In analogy, AM fungal colonization appears to be perceived as a stress for the roots of potato and could be then affecting the sugar partitioning between root and shoot. More biochemical and physiological data would be required to ascertain how the downregulation of such genes affects the sugar allocation to roots.

Eight of the 12 mycorrhiza-induced *SWEET* genes encode proteins from clade I or II that likely transport hexoses (Figures 1, 4B), while most of the repressed genes encode for proteins of clade III, presumed sucrose transporters (Figures 1, 4B). This could be interpreted as an increase in hexoses vs. sucrose being transported across root plant membranes in response to colonization that could serve for fungal nutrition. However, given that SWEETs are bi-directional transporters and that the precise root tissue and subcellular location of each SWEET is not determinable on its sequence basis, more experimental evidence is required. In this sense it is interesting that all five SWEET transporters induced by *Xanthomonas* TAL effectors in rice belong to the clade III (Streubel et al., 2013) and thus presumed sucrose transporters. Eom et al. (2015) suggest that this might be explained by the fact that, in contrast to sucrose, the hexose pools in the cytoplasm are limited and thus, they might be not sufficient to maintain pathogenic growth.

Although, transcriptional inductions and repressions were modest in most cases, it should be taken into account that they were measured in whole root systems, and thus if only happening in colonized areas, a large dilution effect is expected. In general, the extensive transcriptional reprogramming observed involving 22 of the 35 transporters highlights a complex carbohydrate reorganization that roots undergo during mycorrhizal symbiosis. And it also suggests that a large degree of functional redundancy might exist.

### Promoter analysis of potato SWEET transporters

A putative role for the carboxy terminal domain of SWEETs in the posttranslational regulation of their activity has been postulated (Niittylae et al., 2007). However, several lines of evidence have shown that transcriptional regulation might be sufficient to control SWEET activity. Thus, for instance, TAL-mediated SWEET expression induction by pathogens, or overexpression of specific SWEETs is enough to produce significant phenotypic differences (reviewed in Eom et al., 2015). Therefore, and assuming that SWEET genes could be targets for mycorrhiza induction, we next analyzed the promoter of potato *SWEETs* for mycorrhiza-specific motifs (Supplementary Table 3). In particular we searched for the CTTC motif, that has been previously shown to be sufficient for expression in cells containing arbuscules and that is often associated to the phosphate starvation motif P1BS, GnATATnC (Rubio et al., 2001), both in close vicinity to the ATG (Karandashov et al., 2004; Chen et al., 2011; Lota et al., 2013). The core motif of the CTTC element is TCTTGTTC, however, shorter versions lacking the 3′C (TCTTGTT), versions where G is exchanged to C, and a version with both modifications have also been found in mycorrhiza inducible promoters (Lota et al., 2013). Therefore, we also searched for such modifications in the promoter of all potato *SWEET* genes.

The complete CTTC consensus sequence was only found in the promoters of *SWEET7c, 10a*, and *17a*, although only *SWEET17a* was induced by *R. irregularis* at 6 wpi, and in none of the three promoters the P1BS motif was present. Modified versions of the CTTC consensus sequence could be identified in promoters of 28 of the 35 *SWEET* genes, with the exception of *SWEET1c, 1d, 1f*, *1g, 2a, 2b*, and *12f*. The P1BS motif was present in 12 *SWEET* promoters, however it was never in the 30 bp region upstream of the CTTC motif as described (Chen et al., 2011; Lota et al., 2013). We could not identify a clear correlation between the presence of the CTTC motif and a mycorrhiza-induced expression (Supplementary Table 3) and therefore, future deletion experiments will be necessary to determine its importance or not for the mycorrhiza induction of SWEET genes.

**Figure 5:**
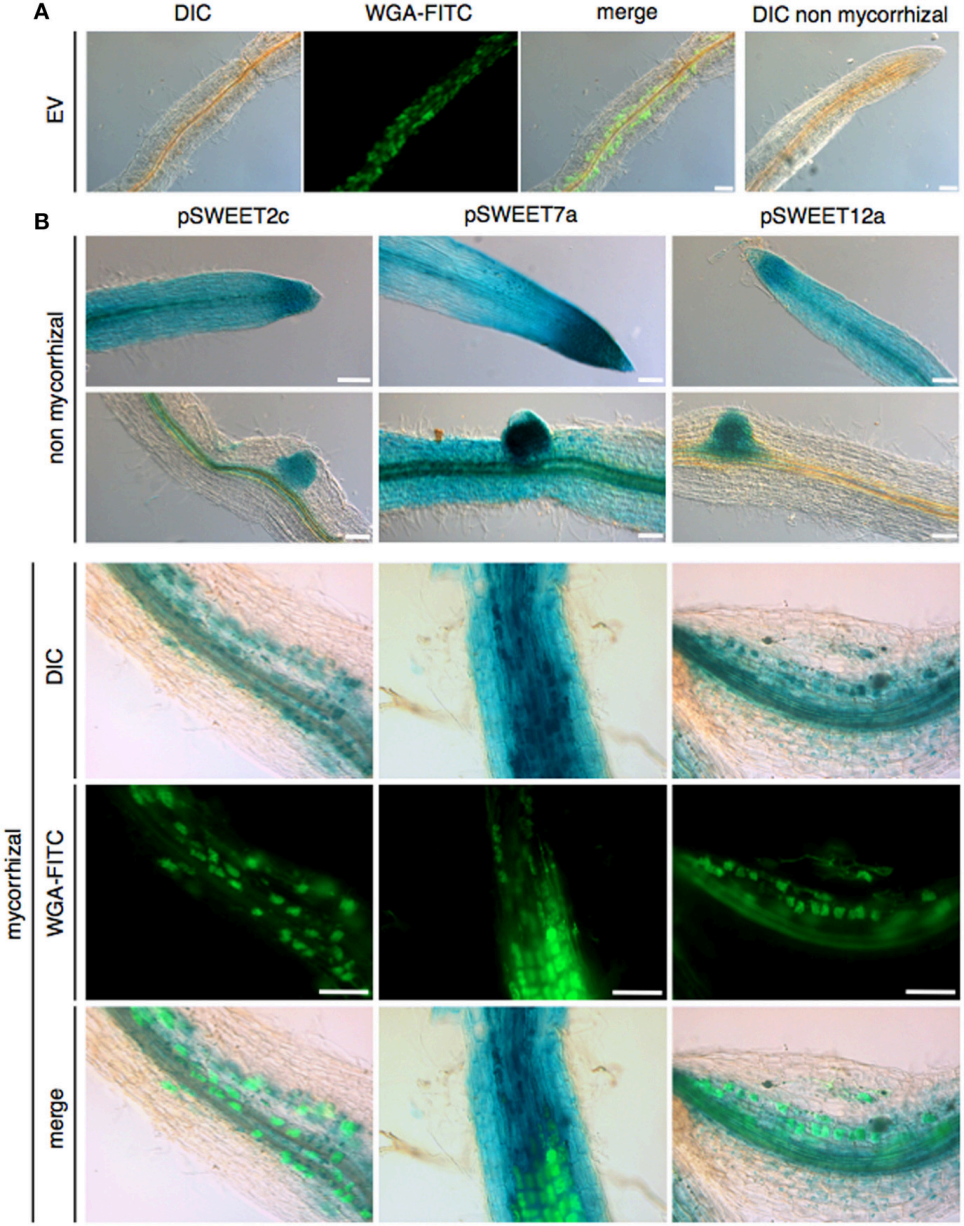
**Promoter-reporter assays of three ***SWEET*** promoters during AM symbiosis**. 2kb fragments upstream of the ATG of potato *SWEETs 2c, 7a*, and *12a* were cloned in front of the GUS reporter gene and transformed in *M. truncatula*. Composite plants were inoculated with *R. irregularis* and harvested 5 wpi (weeks post inoculation). β-Glucuronidase staining and WGA-FITC (wheat germ agglutinin-fluorescein isothiocyanate) counterstaining of fungal cell walls was carried out in mycorrhizal and non-mycorrhizal control roots. **(A)** Empty vector control roots. **(B)** Non-mycorrhizal and mycorrhizal promoter-reporter roots. Scale bars represent 100 μm. DIC: differential interference contrast, WGA-FITC signal is shown in green.

In order to gather information on the spatial expression pattern of mycorrhiza-induced *SWEETs* in roots, promoter-reporter assays using the *GUS* gene were carried out in a heterologous system, in *M. truncatula* transformed with *Agrobacterium rhizogenes*. To that end, three mycorrhiza-induced genes, one from each clade, were selected. The promoter of *SWEET2c* (clade I) showed activity in non-colonized roots mainly at the root tip, as well as faint expression in the cortex. Upon fungal colonization, expression level was increased and re-localized mainly to arbuscule-containing cells (Figure 5). This is in agreement with the presence of several CTTC motifs, two short and one short with a G to C exchange (Supplementary Table 3). Recently, the putative ortholog of SWEET2c from *Arabidopsis*, one of the three highest expressed *SWEETs* in *Arabidopsis* roots, has been functionally characterized (Chen et al., 2015a). Surprisingly, although it belongs to the clade I, AtSWEET2 localizes at the tonoplast and most likely performs as glucose importer. Because AtSWEET2 is mainly localized in root tips, its function has been associated to prevent losses of carbon into the rhizosphere, and consistently, its deletion not only increases glucose efflux but also the plant susceptibility to the pathogenic oomycete *Pythium irregulare* (Chen et al., 2015a). The *Vitis vinifera* putative ortholog, *VvSWEET2a*, is also induced in leaves upon inoculation with *Plasmopara viticola* or with *Botrytis cinerea* (Chong et al., 2014), suggesting that this *SWEET* gene might be a common target of different microorganisms. However, in order to infer if the mycorrhizal induction and re-localization of potato SWEET2c to arbuscule-containing cells would also serve as a mechanism to control sugar efflux toward the AM fungus preventing a parasitic behavior, localization and inactivation studies should be carried out. An alternative hypothesis is that SWEET2c could contribute to maintain the concentration gradient of sucrose in cortical cells by sequestering glucose derived from the activity of cytoplasmic invertases or sucrose synthases. In support of this second scenario, Baier et al. (2010) demonstrated that sucrose synthase was induced in arbuscule containing cells and that its inactivation severely impaired arbuscule development.

Promoter-reporter activity for *SWEET7a*, from clade II, confirmed that although this gene is ubiquitously and highly expressed in the root under non-mycorrhizal conditions (Figure 5), symbiosis induces its expression and this is largely occurring in arbuscule-containing cells that show a much stronger GUS activity than non-colonized cortical cells. This is consistent with the presence of several modified CTTC motifs (Supplementary Table 3). Potato *SWEET7a* does not have an ortholog in tomato, indicating that if it plays a mycorrhiza important role, this function might be redundant with another potato *SWEET*. This is likely, given that most of the up-regulated *SWEETs* during mycorrhiza symbiosis, such as *SWEET7a*, belong to the clades I and II that presumably transport hexoses.

In contrast, only two genes from clade III were induced during AM symbiosis (Figures 1, 4). One of them is *SWEET12a*, whose transcript was induced at 8 wpi, although it was also highly expressed at 6 wpi. GUS activity showed that while in control roots promoter activity was restricted to the root tip, in mycorrhizal roots *SWEET12a* had a strong promoter activity in the cortex, particularly in arbuscule-containing cells (Figure 5). This result is consistent with the presence of two CTTC short motifs in the promoter (Supplementary Table 3). Experiments in progress to delete these motifs will show whether they are required or not for the expression of potato *SWEET12a* in arbuscule-containing cells.

The fact that all three of these promoter-reporter constructs from *SWEETs* induced during mycorrhiza colonization in potato also showed this pattern in the heterologous plant *M. truncatula* supports the idea that the promoter elements and transcription factors driving mycorrhiza and/or arbuscule expression of *SWEETs* must be conserved in both plants. More detailed analyses are now required to identify whether these would be promoter elements only specific to SWEET genes or conserved in other mycorrhiza-induced genes. The results of the promoter-reporter analyses also showed that the induction of *SWEETs* expression was not widely distributed but focused in arbuscule-enriched areas, indicating a redirection of the carbon sink toward colonized cortical cells.

**Figure 6:**
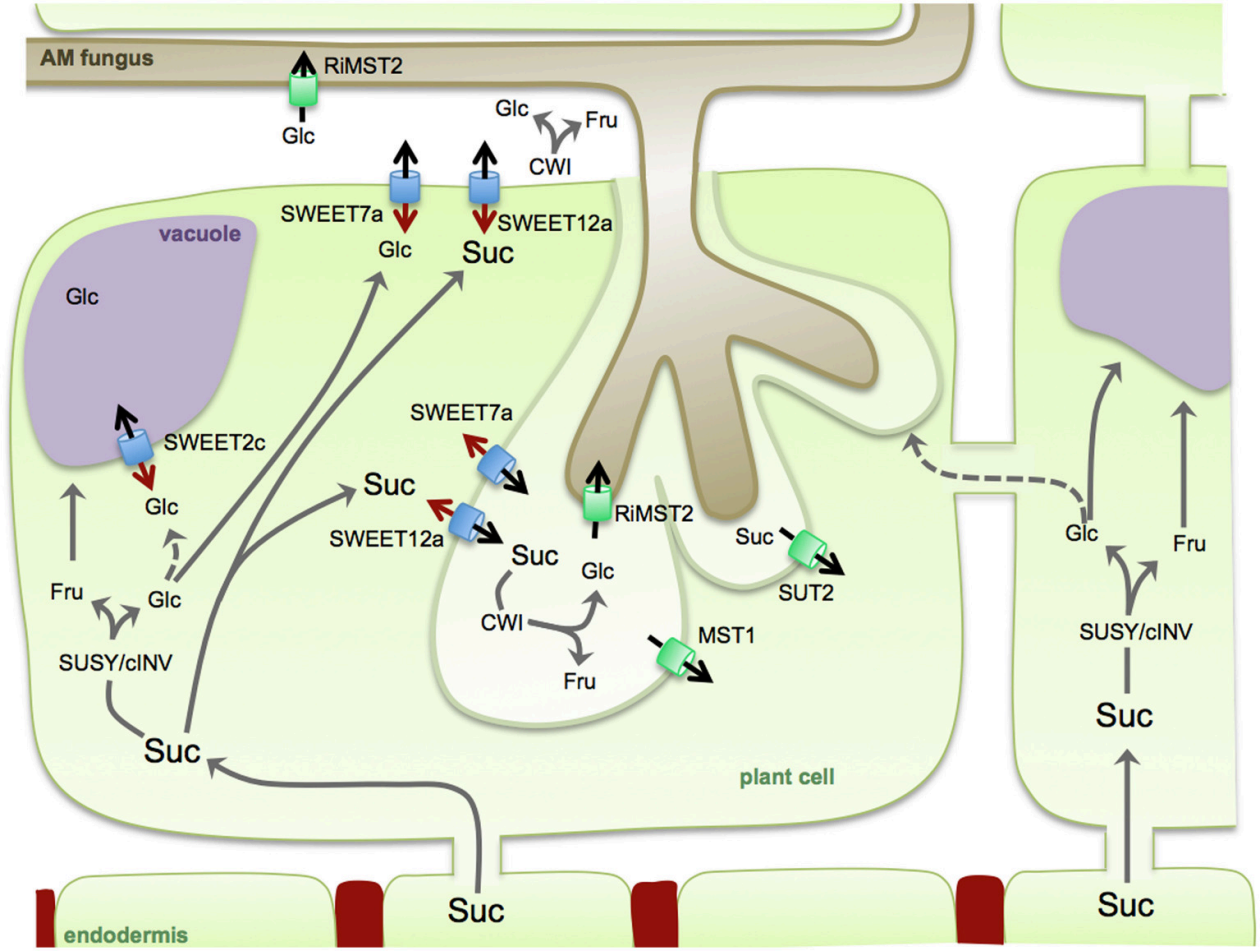
**Model of sugar partitioning during arbuscular mycorrhiza symbiosis**. Sucrose (Suc) is delivered to arbuscule containing cells symplastically from the endodermis to overcome the casparian strip (in red). In the cytoplasm, Suc can be cleaved by sucrose synthase (SUSY) or cytoplasmic invertase (cINV) to glucose (Glc) and fructose (Fru). To maintain the favorable concentration gradient, hexoses could be translocated into the vacuole via tonoplast located SWEETs or other transporters. Alternatively, hexoses could be exported into the apoplast with the help of SWEET7a. Direct export of sucrose into the apoplast or the periarbuscular space could be achieved by sucrose efflux transporters from clade III such as SWEET12a. In the apoplast and periarbuscular space sucrose is cleaved by cell wall-bound invertase (CWI). The sugars in the apoplast are either taken up by the fungus via monosaccharide transporters such as RiMST2 or by the plant cell via monosaccharide transporters such as MST1 (shown in *M. truncatula*) and via sucrose transporters such as SUT2 (shown for *S. lycopersicum*). Neighbor cells might also contribute to the nutrition of the arbuscule containing cell by providing sugars symplastically. Black arrows on SWEETs: sugar transport direction as described above. Red arrows on SWEETs: alternative sugar transport direction.

In conclusion, our work here offers the first overview of the SWEET family in potato, and particularly suggests a putative involvement for some SWEETs during mycorrrhiza symbiosis. The results show that the transport of hexoses across the plant plasma membrane, assuming this is the main role for SWEETs in clade I and II, is highly induced during mycorrhiza formation (eight induced genes), while most repressed genes belong to the clade III (eight genes) and only two induced. If an export activity for SWEET hexose transporters such as SWEET7a would be assumed, these findings would be consistent with the fact that *R. irregularis* induces its monosaccharide transporter MST2 with highest affinity for glucose (Helber et al., 2011). However, the fact that the CW invertase is always expressed in cells containing arbuscules and in cells in contact with intercellular hyphae, suggests that sucrose export into the apoplast is also enhanced during colonization (Schaarschmidt et al., 2006 and our results here). Although, increases in invertase activity in the root do not result in changes in colonization, inhibition of root invertase activity does, suggesting that hexose concentration in the apoplast is not the limiting factor for the symbiosis but that sucrose cleavage in the apoplast is key for the symbiosis (Schaarschmidt et al., 2007a). Altogether, this would be in support for a role of the two induced *SWEETs* from clade III, putative sucrose exporters, during AM symbiosis. Because the cellular localization of all three SWEETs analyzed in mycorrhizal roots points toward an induction in arbuscules, we think that the supply of carbohydrates to the fungus is a complex process involving many different transport processes. As depicted in our model (Figure 6), sucrose reaches the cortex symplastically and it could be directly exported to the periarbuscular space (PAS) and to the apoplast of cells in contact with the fungus with the help of SWEET transporters from clade III, such as SWEET12a, induced by the fungus in arbuscule containing cells. Sucrose in the PAS could then be cleaved with help of the CW invertase, and glucose would be taken up into the fungal cell by the fungal monosaccharide transporter MST2. But which function would then have the induced SWEETs from clade I and II? One possibility is that sucrose could be cleaved in the cytoplasm by sucrose synthase and/or cytoplasmic invertase. Both activities have been shown in arbuscule containing cells and they could help to maintain the concentration gradient. Glucose could then be further exported in the PAS by some of the induced SWEETs from clade I and II. Vacuolar SWEETs such as the induced SWEET2c could also contribute to maintain the favorable sucrose gradient in arbuscule containing cells by sequestering glucose and/or fructose in the vacuole.

Alternatively, mycorrhiza induced SWEETs from clade I and II might be functioning not as exporters but rather as importers, competing with the fungus at the apoplast for the re-import of the sugar into the plant cytoplasm to support the high metabolic activity of the colonized cells or to avoid defense responses elicited by high amount of hexoses (Schaarschmidt et al., 2007b). In support of that, other transporters have been identified featuring in arbuscule containing cells to re-import sugar from the apoplast, including a monosaccharide transporter in *M. truncatula* and a sucrose transporter in tomato (Harrison, 1996; Bitterlich et al., 2014). More studies, in particular regarding the biochemistry, subcellular localization, and physiology of the potato SWEETs regulated during symbiosis are required to be able to draw a more precise model of their role in sugar partitioning during mycorrhizal symbiosis.

## Author contributions

NR, JM planned the experiments and wrote the manuscript, JM carried out the experiments.

### Conflict of interest statement

The authors declare that the research was conducted in the absence of any commercial or financial relationships that could be construed as a potential conflict of interest.

## Supplementary material

Supplementary Figure 1 **Topology analysis of potato SWEETs**. The upper panel shows the predicted domain structure of potato SWEETs in the NCBI database that were predicted to have more or less than 7 TM domains. Manual annotation and expression data (here not shown) revealed that almost all of them were wrongly annotated. Only *SWEET7c* that is possibly a pseudogene contains only 4 TM domains.

Supplementary Table 1 **Accession numbers of sequences used in this study and genome positions and transmembrane domain prediction for potato SWEETs**.

Supplementary Table 2 **Primers used in this study**.

Supplementary Table 3 **Promoter element analysis of potato and tomato SWEETs**.

Supplementary Table 4 **Identity matrices among potato and between potato and tomato SWEETs**.

Supplementary Table 5 **RNA-seq data for annotated potato SWEETs from the Spud DB**.
