# Supplementary material for: Arbuscular mycorrhiza Symbiosis Induces a Major Transcriptional Reprogramming of the Potato SWEET Sugar Transporter Family
Source: Front Plant Sci. 2016 Apr 14;7:487. doi: 10.3389/fpls.2016.00487 (PMC4830831; doi:10.3389/fpls.2016.00487)
Supplement: Supplementary Figure 1 — Topology analysis of potato SWEETs. The upper panel shows the predicted domain structure of potato SWEETs in the NCBI database that were predicted to have more or less than 7 TM domains. Manual annotation and expression data (here not shown) revealed that almost all of them were wrongly annotated. Only SWEET7c that is possibly a pseudogene contains only 4 TM domains. [file Presentation1.PPTX]

## Slide 1
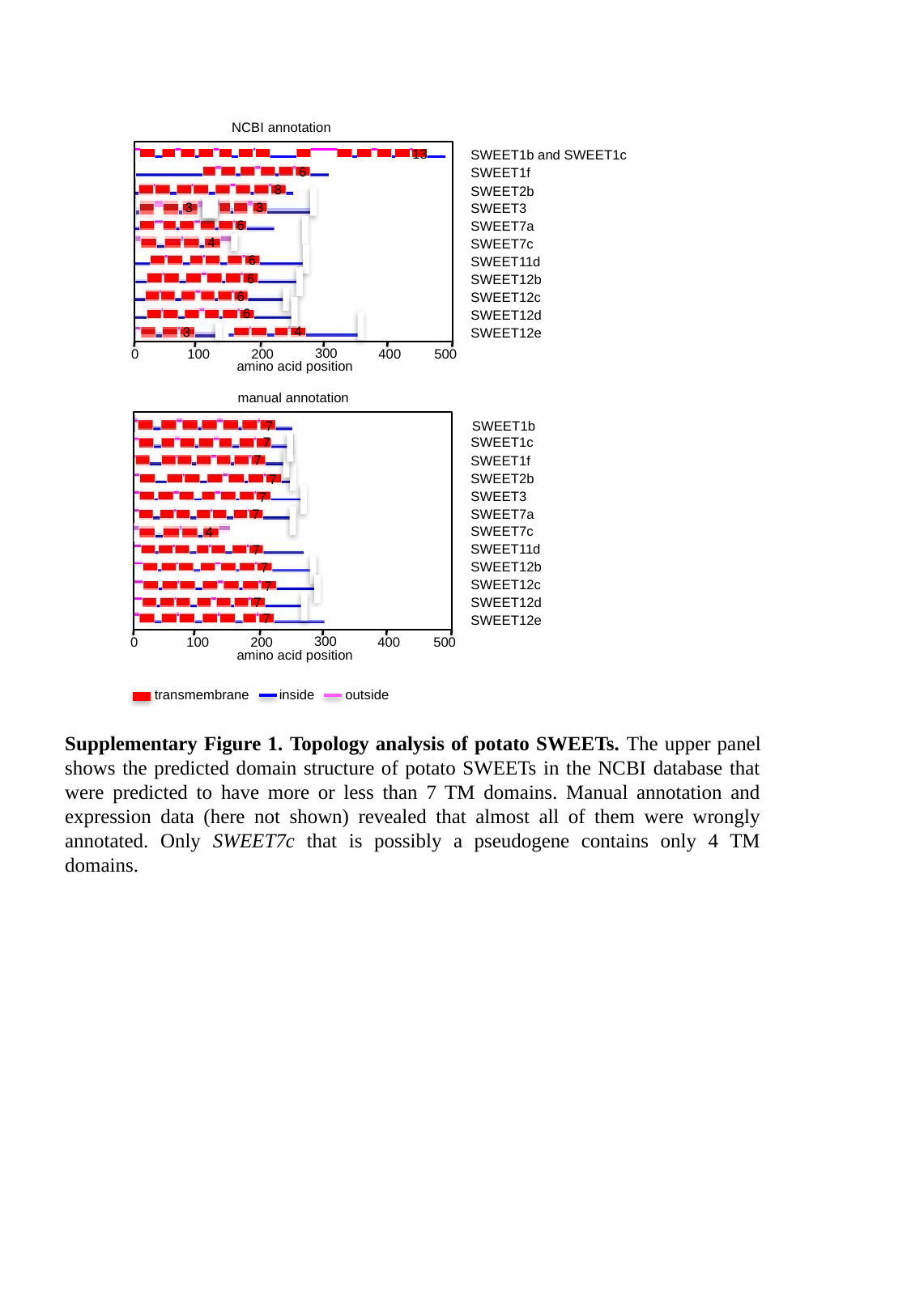

NCBI annotation
13
SWEET1b and SWEET1c
SWEET1f
SWEET2b
SWEET3
SWEET7a
SWEET7c
SWEET11d
SWEET12b
SWEET12c
SWEET12d
SWEET12e
6
8
3
3
6
4
6
6
6
6
4
3
300
0
100
200
400
500
amino acid position
manual annotation
SWEET1b
SWEET1c
SWEET1f
SWEET2b
SWEET3
SWEET7a
SWEET7c
SWEET11d
SWEET12b
SWEET12c
SWEET12d
SWEET12e
7
7
7
7
7
7
7
7
7
7
7
300
0
100
200
400
500
amino acid position
transmembrane
inside
outside
4
Supplementary Figure 1. Topology analysis of potato SWEETs. The upper panel shows the predicted domain structure of potato SWEETs in the NCBI database that were predicted to have more or less than 7 TM domains. Manual annotation and expression data (here not shown) revealed that almost all of them were wrongly annotated. Only SWEET7c that is possibly a pseudogene contains only 4 TM domains.
